# RELATION OF SPECIFIC DOMAINS OF AGING KNOWLEDGE AND CONTACT TO AGEIST ATTITUDES AMONG DOCTORAL PSYCHOLOGY STUDENTS

**DOI:** 10.1093/geroni/igad104.2708

**Published:** 2023-12-21

**Authors:** Grace Caskie, Benjamin Johnson, Eve Root

**Affiliations:** Lehigh University, Bethlehem, Pennsylvania, United States; Lehigh University, Bethlehem, Pennsylvania, United States; Lehigh University, Bethlehem, Pennsylvania, United States

## Abstract

Knowledge about aging and intergenerational contact were identified in Levy’s (2018) Positive Education about Aging and Contact Experiences (PEACE) model as the two elements that are key to reducing ageist attitudes. Existing empirical literature examining these relationships has primarily focused on undergraduate, rather than graduate, students and has typically operationalized knowledge about aging as a unidimensional construct (i.e., total score). Thus, in this study, we examined how knowledge within three specific domains of aging (psychological, social, biological) as well as quantity of contact with older adults related to ageist attitudes among doctoral psychology students (N=192; age=21-58 years). Participants completed the Contact with Older Adults Scale, Facts about Aging Quiz, Fraboni Scale of Ageism (subscales: Stereotypes, Separation, Affective Attitudes), and Ambivalent Aging Scale (subscales: Hostile Ageism, Benevolent Ageism). Controlling for students’ age and aging coursework, the three knowledge domains and contact together explained between 8%-24% of the variance in the five measures of ageist attitudes. As expected, greater knowledge of psychological aging related to less endorsement of ageist stereotypes and less separation from older adults; however, greater knowledge of biological aging related to more ageist attitudes across all five measures, and greater knowledge of social aging related to more benevolent ageist attitudes. More contact with older adults related to less separation and less negative affect about older adults, but not to the other ageist attitudes measures. Differentiating between domains of aging knowledge produced unique relations with both explicitly negative and ambivalent ageist attitudes, which ageism intervention designers may need to consider.

